# Macrophage Migration Inhibitory Factor as an Emerging Drug Target to Regulate Antioxidant Response Element System

**DOI:** 10.1155/2017/8584930

**Published:** 2017-01-16

**Authors:** Hiroshi Yukitake, Masayuki Takizawa, Haruhide Kimura

**Affiliations:** Pharmaceutical Research Division, Takeda Pharmaceutical Company Ltd., 26-1 Muraoka-Higashi 2-Chome, Fujisawa, Kanagawa 251-8555, Japan

## Abstract

Oxidative stress is involved in pathophysiology and pathological conditions of numerous human diseases. Thus, understanding the mechanisms underlying the redox homeostasis in cells and organs is valuable for discovery of therapeutic drugs for oxidative stress-related diseases. Recently, by applying chemical biology approach with an ARE activator, BTZO-1, we found macrophage migration inhibitory factor (MIF) as a new regulator of antioxidant response element- (ARE-) mediated gene transcription. BTZO-1 and its active derivatives bound to MIF and protected cells and organs from oxidative insults via ARE activation in animal models with oxidative stress such as ischemia/reperfusion injury, inflammatory bowel diseases, and septic shock. In this review, we briefly highlight key findings in understanding the MIF-ARE system.

## 1. Introduction

Reactive oxygen species (ROS) and reactive nitrogen species (RNS) play a dual role, beneficial and deleterious functions, in living systems [1, 2]. ROS at low or moderate physiological concentrations mainly work as important signal mediators in multiple systems, including cellular defensive response and induction of a mitogenic response [1]. Excessive level of ROS/RNS, that is, oxidative stress and nitrosative stress, is harmful and damages cell structures, including lipids and membranes, proteins, and DNA [1, 3, 4]. Overproduction of ROS occurs due to dysregulation of mitochondrial electron transport chain or excessive stimulation of NAD(P)H. Oxidative stress and nitrosative stress are associated with imbalance between production of ROS and a function of enzymatic/nonenzymatic antioxidant reaction system in living organisms, and disturbance of this homeostasis plays a critical role in pathophysiology of human diseases, such as cardiovascular diseases, inflammatory bowel diseases, septic shock, rheumatoid arthritis, Alzheimer's disease, Parkinson's disease, multiple sclerosis, amyotrophic lateral sclerosis, schizophrenia, ischemia/reperfusion, atherosclerosis, diabetes mellitus, cancer, and other diseases, and in aging [1, 5–12]. Therefore, regulation of redox homeostasis would be a promising approach in the treatment and/or prevention of these diseases.

Antioxidant response element (ARE) is one of the control batteries for redox homeostasis. Induction of ARE-regulated gene expression is an intrinsic defense system and decreases oxidative stress in cells and organs [13–16]. ARE is a cis-acting DNA regulatory element located in the regulatory regions of multiple genes encoding phase II detoxifying enzymes and cytoprotective proteins, including glutathione S-transferases (GSTs), heme oxygenase-1 (HO-1), reduced nicotinamide adenine dinucleotide phosphate (NAD(P)H), quinone oxidoreductases (NQOs), UDP-glucuronosyl transferase (UDP-GT), epoxide hydrase, *γ*-glutamylcysteine synthetase (*γ*-GCS), and peroxiredoxin 1 [13–17]. Thus, activation of the ARE is of critical importance to cellular protection against oxidative stress and could be a therapeutic target for oxidative stress-related diseases.

## 2. Macrophage Migration Inhibitory Factor (MIF) as a Key Regulator of Antioxidant Response Element (ARE) System

ARE is an enhancer element having the consensus sequence TGACnnnGC [17, 18]. Many studies have supported the hypothesis that activation of ARE-mediated gene expression is mainly regulated by the transcription factor Nrf2, a member of the cap'n'collar family of basic region-leucine zipper transcription factor [19–22]. Nrf2 is a cytoplasmic protein sequestered by direct binding with the actin-bound protein Keap1 (Kelch ECH associating protein), a Cul3-based E3 ligase [23–25]. Under normal conditions, Nrf2 protein, via direct binding with Keap1, is strictly maintained at low levels by ubiquitination and consequent 26S proteasome degradation [23–29]. Under some pathological conditions, oxidative factors dissociate Keap1-Nrf2 complex, and that leads to increase in Nrf2 protein levels and nuclear translocation of Nrf2. Nrf2 in nucleus dimerizes with small Maf proteins and binds to the ARE, resulting in expression of many phase II detoxyfying and cytoprotective genes [30, 31]. Thus, Keap1-Nrf2 system is an attractive target for the induction of ARE-mediated gene expression.

We recently identified macrophage migration inhibitory factor (MIF) as a key regulator of ARE-mediated gene expression by chemical biology approach using BTZO-1, a 1,3-benzothiazin-4-on derivative, as chemical probe [32]. MIF, also named as glycosylation-inhibiting factor or phenylpyruvate tautomerase, was originally identified as a soluble factor with macrophage migration-inhibiting properties in the culture medium of activated T lymphocytes [33–37]. MIF has been considered as a cytokine regulating innate and acquired immune responses [37–39]. However, molecular behavior and expression pattern of MIF are different from those of conventional cytokines. For example, MIF is produced by a variety of cells, such as monocytes, macrophages, granulocytes, lymphocytes, eosinophils, neutrophils, endocrine cells, epithelial cells, endothelial cells, and smooth muscle cells, and exists as ubiquitous protein both intra- and extracellular [37, 40–46]. MIF has been reported to have a wide variety of other biological functions, such as counterregulation of glucocorticoid in inflammation, negative regulation of p53-mediated growth arrest and apoptosis, and activation of component of the mitogen-activated protein kinase and Jun-activation domain-binding protein-1 (Jab-1) pathway [47–50]; however, its precise function in the majority of cells is not known.

BTZO-1 was originally discovered from our chemical library as a cardiomyocyte-protective agent; BTZO-1 protected rat primary cardiomyocyte from serum deprivation-induced cell death [32]. Investigation of the mode of action of BTZO-1 revealed that BTZO-1 and its active derivatives activated ARE-mediated gene expression. Drug-affinity chromatography and surface plasmon resonance (SPR) biosensor technique showed that BTZO-1 and its active derivatives in both protection of rat primary cardiomyocyte from serum deprivation-induced cell death and ARE activation directly and selectively bound to MIF [32]. The structure-activity relationship of BTZO-1 derivatives in binding to MIF agreed well with that in ARE-mediated gene expression, as well as cardioprotection. Thus, MIF was considered as a molecular target of BTZO-1. In line with this hypothesis, recombinant purified MIF protein induced ARE-mediated gene expression and suppressed nitric oxide- (NO-) induced cardiomyocyte death in vitro [32]. Furthermore, BTZO-1 promoted MIF-induced ARE activation in H9c2 cells, while BTZO-1-induced ARE activation was decreased in the MIF-reduced H9c2 cells transfected with MIF siRNA [32]. The reduction of ARE-mediated gene expression by BTZO-1 in the MIF-reduced H9c2 cells was restored by applying recombinant MIF to the culture medium. Although the precise intracellular signaling pathway to activate ARE-mediated gene expression after BTZO-1-MIF interaction is unknown, our chemical biological approach with BTZO-1 derivatives suggested that MIF has a pivotal role in ARE-mediated gene regulation [32].

Recently, it was reported that MIF antagonized apoptosis induced by cigarette smoke, a generator of oxidative stress, in human pulmonary endothelial cells, and MIF knockout mice potentiated the toxicity of cigarette smoke exposure via increased apoptosis of endothelial cells [51]. Another study demonstrated that MIF expression levels and cellular antioxidant activity levels were age-dependently decreased in lung, and the analysis with MIF knockout mice revealed that the reductions in MIF expression levels contribute to age-related radiation-induced lung injury in mice [52], and the decrease in MIF appears to lead to the dysregulation of Nrf2, antioxidant activities, and Nrf2 nuclear concentrations [52]. Moreover, another group demonstrated that MIF provided cardioprotection during ischemia/reperfusion by reducing oxidative stress [53].

These studies support our discovery that MIF works, at least in part, as an upstream signal node for ARE-mediated gene transcription.

## 3. Is MIF a Sensor for ARE Activation?

ARE is also named electrophile/xenobiotic response element, and ARE-mediated gene expression is activated by not only oxidative stress but also electrophilic molecules and heavy metals [17, 25]. Keap1 is also known as a sensitive sensor not only for oxidative stress but also for xenobiotics such as electrophilic molecules in cytosol [15, 16, 20–28]. Keap1 is a cysteine-rich protein and its cysteine residues have essential roles in Keap1-dependent ubiquitination of Nrf2 [54, 55]. Oxidative/electrophilic molecules modified these cysteine residues, and these modifications resulted in disruption of efficient ubiquitination of Nrf2 and Nrf2 degradation by proteasome. Following the saturation of Keap1 by dysregulation of degradation of Nrf2, newly synthesized Nrf2 proteins accumulate within the cell and translocate to the nucleus, leading to ARE activation [15, 16, 20–28, 54, 55].

Our binding assays using both wild type and mutant MIFs demonstrated that BTZO-1-MIF interaction required the intact N-terminal Pro1 in MIF [32]. Interestingly, Pro1 is nucleophilic due to its location in a hydrophobic environment and has been reported to interact with electrophiles and alkylating agents [56, 57]. In fact, some of the known ARE activators, such as 15-deoxy-Δ12,14-prostaglandin J2 (15d-PGJ2) a lipid-derived electrophilic molecule, bound to Pro1 site in MIF in the scintillation proximity assay using radio-labeled BTZO-1 as a ligand. Known ARE activators with binding affinity to MIF might activate ARE via binding to MIF [32]. Based on these observations, we propose a hypothesis that MIF N-terminal Pro1 domain functions as a sensor for deleterious electrophiles. Further studies to investigate this hypothesis are worth trying.

## 4. Potential of MIF-ARE System as Therapeutic Target

Disturbance of redox homeostasis plays a critical role in pathophysiology of several human diseases [1, 5–12]. MIF seems to be a sensor for oxidative stress and/or upstream regulator for ARE as described above; thus, MIF-ARE system may become a new therapeutic target for many diseases caused by excessive oxidative stress. In fact, our recent preclinical studies demonstrated that BTZO-2, a BTZO-1 analog with a better ADME profile, protected heart tissues during ischemia/reperfusion injury in rats [32]. BTZO-2 also protected lipopolysaccharide-induced endotoxic shock in mice [58]. BTZO-15, another active BTZO-1 derivative, ameliorated chemically induced colitis in rats [59]. In addition, 15d-PGJ2, which showed MIF binding affinity in a scintillation proximity assay (SPA) [32], exerts anti-inflammatory activity through activation of ARE and suppressed carrageenan-induced pleurisy [60]. Further screenings for ARE activators via MIF are worthwhile, and MIF-ARE system may be a high-value therapeutic target for wider oxidative stress-related diseases beyond Nrf2-ARE system.

## 5. Development of ARE Activators as Therapeutic Drugs

**Figure 1:**
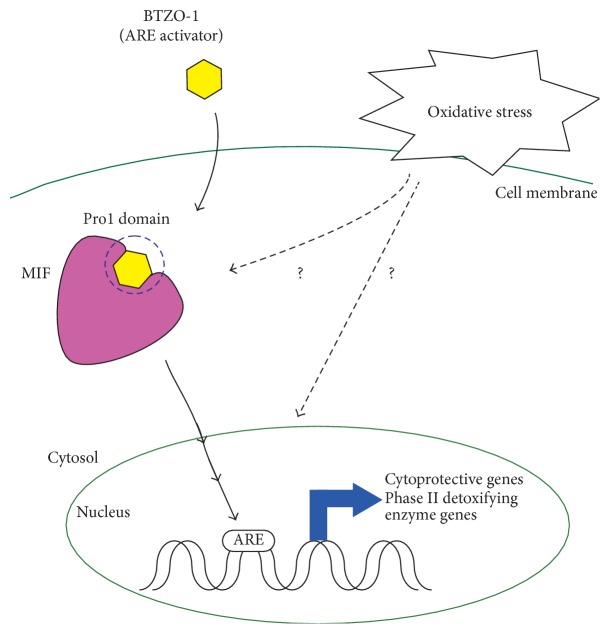
BTZO-1 induces ARE-mediated gene expression via MIF under oxidative conditions. MIF has nucleophilic part around N-terminal Pro1 region and BTZO-1 bound to the Pro1 region.

As discussed, induction of ARE-mediated gene expression is promising as a therapeutic target. However, there are few ARE activators with acceptable safety profiles for therapeutic drugs. ARE system is an intrinsic system against oxidative stress and/or xenobiotics; thus, toxic compounds might show strong efficacy in some drug screening campaigns aiming for ARE-transcriptional activator. These “false-positive toxic compounds” may hinder discovery of safer ARE activators. Therefore, new approaches and/or breakthrough for discovery of safer ARE activators are needed. BTZO-1 was identified in a cell-based and phenotypic screening program aiming for cardioprotective agents. Interestingly, BTZO-1 induced ARE-mediated gene expression without exhibiting cytotoxicity. One unique feature of BTZO-1 is that it activated ARE-mediated gene induction only under oxidative stress conditions, but not under normal conditions (Figure 1) [32]. It is unclear why BTZO-1 induced ARE activation only under the oxidative stresses conditions, but this aspect of the unique profile of BTZO-1 could provide a novel avenue for understanding regulation of ARE activation and also for discovery of safer ARE activators. Furthermore, MIF-ARE activators like BTZO-1 may have different pharmacodynamics (PD) profiles compared with Keap1-modifying inducers of Nrf2. The difference in PD profiles will depend on Keap1 and MIF expression patterns and their signal contribution in the target tissues for therapy. And if MIF and Keap1 may capture different stresses/ligands, it may also lead to different PD profiles.

## 6. Future Directions

Clinical development of ARE activators as therapeutic drugs is an active area. For example, dimethyl fumarate (DMF) (Tecfidera™), the effects of which are believed, at least in part, to be mediated via Nrf2-ARE system, has been approved by the U.S. Food and Drug Administration as a therapy for multiple sclerosis [61]. CDDO-Me (2-cyano-3,12-dioxooleana-1,9 (11)-diene-28-oic acid methyl ester), also named as bardoxolone methyl, has been clinically studied against a variety of disorders [62]. Although phase III trials of CDDO-Me for chronic kidney disease in the USA failed because of a high rate of cardiovascular adverse events in subpopulation of susceptible patients with an increased risk for heart failure at baseline [63], several clinical trials, such as for treatment of pulmonary hypertension in the US and for chronic kidney disease associated with type 2 diabetes in Japan, are still ongoing. In cancer, the Nrf2-ARE system has emerged as a new therapeutic target [21, 22]. It was reported that Nrf2-ARE system was constitutively activated in some solid tumors, such as lung cancer and esophageal carcinoma, and it contributed to unfavorable prognosis [21, 64–66]. It was traditionally thought that the cancer cells took over and utilized Nrf2-ARE system for survival and malignant growth [21, 64–66]. However, recent reports suggested that Nrf2-ARE activation by CDDO-Me abrogates the immune-suppressive effects of myeloid-derived suppressor cells (MDSCs) and improves immune responses in cancer patients, and Nrf2 activation in MDSCs prevents cancer cell metastasis [22, 67–71]. There seems to be room to apply ARE activator to cancer therapy. Some reviews and papers have also pointed out that targeting the Nrf2-ARE system is a promising strategy to tackle neurodegenerative disorders [16, 19, 25, 72].

Here, we propose that MIF could be a critical regulator of ARE-mediated gene expression. Quite interestingly, BTZO-1 activated the ARE-mediated gene expression under oxidative stress conditions without showing cytotoxicity. This unique profile of BTZO-1 may open the door for the discovery of new and safer ARE activators as therapeutic drugs for multiple disorders. Drug screening campaigns using radio-labeled BTZO-1 and MIF protein are worth trying to identify novel MIF binders with potential to activate ARE-mediated transcription. Further studies using BTZO-1 derivatives will be needed to narrow down the best indications for ARE activators using the MIF-ARE system.
